# P-144. Antimicrobial resistance to ceftriaxone and ciprofloxacin is increasingly observed in Salmonella Infantis isolates from clinical and retail meat sources in the United States

**DOI:** 10.1093/ofid/ofaf695.371

**Published:** 2026-01-11

**Authors:** Brendan J Kelly, Sameh W Boktor, Edward G Dudley, Nkuchia M M’ikanatha

**Affiliations:** Assistant Professor of Medicine Infectious Diseases at the Hospital of the University of Pennsylvania, Philadelphia, Pennsylvania; Pennsylvania Department of Health |, Harrisburg, PA; Penn State College of Agricultural Sciences, University Park, Pennsylvania; Pennsylvania Department of Health, HARRISBURG, Pennsylvania

## Abstract

**Background:**

Nontyphoidal *Salmonella* (NTS), a leading cause of bacterial foodborne infections in the United States, is typically acquired from contaminated meat. Antimicrobial resistance in NTS varies by serotype. *S.* Infantis is one of the top ten most frequently isolated NTS and resistance to first-line antibiotics is emerging in this serotype. The National Antimicrobial Resistance Monitoring System (NARMS) tracks *Salmonella* from humans and retail meats nationwide.

**Figure:**
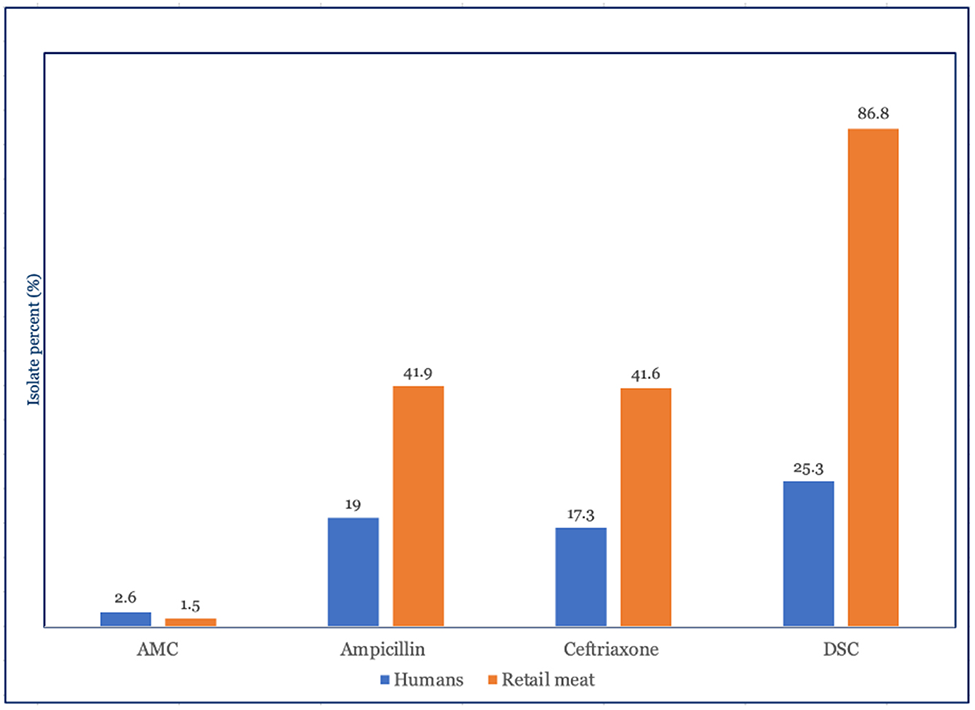
Figure. Percentages of isolates resistant to amoxicillin-clavulanate (AMC), ampicillin, ceftriaxone or with decreased susceptibility to ciprofloxacin (DSC) in patient (blue) and meat (orange) samples.

**Methods:**

We analyzed whole genome sequences and antimicrobial susceptibility profiles for each *S.* Infantis isolate from two NARMS programs: 1. CDC--clinical isolates submitted by public health laboratories; 2. FDA--food isolates identified by collaborating laboratories through prospective testing for *Salmonella* in retail meats.

**Results:**

Among 620 *S*. Infantis isolates from patients during 2012-2023, 157 (25.3%) had decreased susceptibility to ciprofloxacin (DSC) and 107 (17.3%) were ceftriaxone-resistant. Resistance to ampicillin was found in 118 (19.0%) isolates, to amoxicillin-clavulanate in 16 (2.6%), and to azithromycin in two (0.3%). Genetic determinants: 93.5% (100/107) of ceftriaxone-resistant isolates carried extended-spectrum β-lactamase (ESBL) genes [*bla*_CTX-M-65,_ n=86] and [*bla*_CMY−2,_ n=14]; 99.3% (156/157) of isolates with DSC had *gyrA* mutations.

Among 963 *S.* Infantis isolates from retail meat sources during 2002-2021, 836 (86.9%) demonstrated DSC and 401 (41.6%) were ceftriaxone-resistant. Resistance to ampicillin was found in 403 (41.9%) isolates and to amoxicillin-clavulanate in 14 (1.5%). Genetic determinants: 99% (397/401) of ceftriaxone-resistant isolates carried ESBLs [*bla*_CTX-M-65,_ n=384] and [*bla*_CMY−2,_ n=13]; 99.8% (834/836) of isolates with DSC had *gyrA* mutations.

While detected in < 1% of *S.* Infantis in 2012, most recently ESBLs *bla*_CTX-M-65_ were found in 16.9% (11/65 in 2021) of isolates from clinical samples and 47.7% (91/213 in 2023) from retail meats.

**Conclusion:**

Genetic analysis shows that *S.* Infantis isolates from patients, and more frequently from retail meat, harbor ESBL and/or *gyrA* mutations. Resistance to first-line therapies (ceftriaxone and/or ciprofloxacin) in this particular *Salmonella* serotype is emerging as a therapeutic challenge.

**Disclosures:**

All Authors: No reported disclosures

